# DTI and MTR Measures of Nerve Fiber Integrity in Pediatric Patients With Ankle Injury

**DOI:** 10.3389/fped.2021.656843

**Published:** 2021-09-29

**Authors:** Scott A. Holmes, Anastasia Karapanagou, Steven J. Staffa, David Zurakowski, Ronald Borra, Laura E. Simons, Christine Sieberg, Alyssa Lebel, David Borsook

**Affiliations:** ^1^Center for Pain and the Brain, Boston Children's Hospital, Boston, MA, United States; ^2^Department of Anesthesiology, Critical Care and Pain Medicine, Boston Children's Hospital and Harvard Medical School, Boston, MA, United States; ^3^Department of Radiology, University Medical Center Groningen, University of Groningen, Groningen, Netherlands; ^4^Department of Nuclear Medicine and Molecular Imaging, University Medical Center Groningen, University of Groningen, Groningen, Netherlands; ^5^Department of Anesthesia, Stanford University, Stanford, CA, United States; ^6^Departments of Psychiatry and Radiology, Massachusetts General Hospital, Boston, MA, United States

**Keywords:** peripheral nerve imaging, neuropathic pain, pain, ankle sprain, nerve injury

## Abstract

Acute peripheral nerve injury can lead to chronic neuropathic pain. Having a standardized, non-invasive method to evaluate pathological changes in a nerve following nerve injury would help with diagnostic and therapeutic assessments or interventions. The accurate evaluation of nerve fiber integrity after injury may provide insight into the extent of pathology and a patient's level of self-reported pain. The aim of this investigation was to evaluate the extent to which peripheral nerve integrity could be evaluated in an acute ankle injury cohort and how markers of nerve fiber integrity correlate with self-reported pain levels in afferent nerves. We recruited 39 pediatric participants with clinically defined neuropathic pain within 3 months of an ankle injury and 16 healthy controls. Participants underwent peripheral nerve MRI using diffusion tensor (DTI) and magnetization transfer imaging (MTI) of their injured and non-injured ankles. The imaging window was focused on the branching point of the sciatic nerve into the tibial and fibular division. Each participant completed the Pain Detection Questionnaire (PDQ). Findings demonstrated group differences in DTI and MTI in the sciatic, tibial and fibular nerve in the injured ankle relative to healthy control and contralateral non-injured nerve fibers. Only AD and RD from the injured fibular nerve correlated with PDQ scores which coincides with the inversion-dominant nature of this particular ankle injuruy cohort. Exploratory analyses highlight the potential remodeling stages of nerve injury from neuropathic pain. Future research should emphasize sub-acute time frames of injury to capture post-injury inflammation and nerve fiber recovery.

## Introduction

Persistent nociceptive signaling from peripheral nerve fibers can drive pain chronification (1) and significant reductions in quality of life (2). Injury or disease to pain pathways, occurring either peripherally or centrally, are defining features of neuropathic pain (3) and result in diffuse changes throughout the body and brain (4). Importantly, the symptoms of allodynia and hyperalgesia in persons with neuropathic pain (5) highlight the potential of focal nerve-related changes that may be a source of augmented nociceptive drive. It remains unclear the extent to which peripheral nerve fiber integrity is compromised in persons with neuropathic pain from ankle injury and how this is related to self-report levels of pain.

Trauma and disease to peripheral afferent pain fibers compromises nerve fiber integrity. Small fiber neuropathies can be evaluated using a range of techniques; however, there is no current gold standard for diagnosis (6). Current methods for quantitative evaluation of peripheral afferent pain fibers include electromyography and microneurography (7) or skin biopsy (8) for small nerve fibers. These methods require significant time and skill on the part of the evaluator and are not necessarily objective in nature. Nerve trauma to the axons (9) and also overlaying epineurium, and internal perineurium of myelin sheaths may be critical factors in the acute to chronic transition of neuropathic pain (10). Recent advances in magnetic resonance neurography reflect a promising role for diffusion tensor imaging (DTI) and magnetization transfer imaging (MTI). In particular, DTI reflects a standardized and reproducible approach that can be translated to the clinic (11). Investigations evaluating DTI in the peripheral nerve have shown strong reliability as well as reproducibility (12). Clinically, use of DTI in peripheral nerve fibers have shown changes after crush injury and traction showing significant changes in FA measures (13). Variability in MTI is associated with changes in myelin levels (14) and has been used in spinal cord imaging (15) and peripheral nerve (16). The use of peripheral neurography represents a non-invasive and sensitive measure for evaluating nerve fiber integrity.

Stretch injuries associated with ankle sprains can result in neuropathic pain because damage results in nerve traction or a hematoma in the epineural sheath (17). Following ankle sprain, 20–80% of patients have an ankle injury that involves the tibial or fibial (18) divisions of the sciatic nerve. While most recover in the months following the injury, 10–30% continue to have neuropathic pain over time (19). To date, it is unclear to what extent structural changes in peripheral nerves can be observed after an ankle injury, and the extent to which they correlate with self-reported levels of pain. In this study we sought to evaluate the extent to which (i) differences in peripheral nerve integrity could be evaluated using DTI and MTR neurography in persons with neuropathic pain from ankle injury, (ii) changes in nerve fiber integrity could be observed in ipsilateral and contralateral nerve divisions (sciatic, tibial, fibular), and (iii) nerve fiber integrity is correlated with self-reported pain ratings. We elected to explore the impact of sex and time since injury in study findings. We hypothesized that altered nerve integrity as measured by fMRI would provide an objective correlate of the nerve damage from the ankle injury; that such changes that would parallel the patients subjective measures and be a useful marker for treatment efficacy; and that subclinical changes (that may be more sensitive to reinjury) could be seen over time.

## Methods

### Human Subjects

The study was approved by the ethics board at the Boston Children's Hospital and subject experimentation was consistent with human pain studies noted in the Declaration of Helsinki. This study recruited participants from Boston and surrounding areas and was part of a larger investigation evaluating central and peripheral nervous system changes in pediatric subjects with neuropathic pain. A total of 39 patients with lower extremity pain and 16 age/sex-matched healthy controls were recruited. Inclusion criteria was otherwise healthy individuals ages 10–24 who present with a unilateral lower extremity injury and evidence of neuropathic pain as confirmed through medical evaluation by a project-affiliated physician. Exclusion criteria included: claustrophobia, significant medical problems (e.g., uncontrolled asthma, seizures, cardiac disorder), drug use (e.g., opioids, marijuana), psychiatric problems (e.g., active suicidality), and other neurological disorders, pregnancy and any device or medical concern that would preclude an individual being scanned using an MRI (e.g., metalic implant, exceeding weight limit of scanner). Patients were recruited from the Boston Children's Hospital Division of Sports Medicine, Emergency Department, and Department of Orthopedic Surgery. They were contacted about the study through participating physicians (AL) during their clinical appointment or a member of the research team *via* a letter that was mailed home along with an opt-out card. Healthy controls were recruited from the Boston community through advertisements and postings. All participants were compensated for their time. A detailed neurological examination was performed to ensure that subjects were otherwise healthy. All participants underwent a neurological evaluation as part of intake and were administered the Pain Detection Questionnaire [PDQ; (20)] at the time of study visit to determine the level of pain reporting.

### Imaging

**Figure 1:**
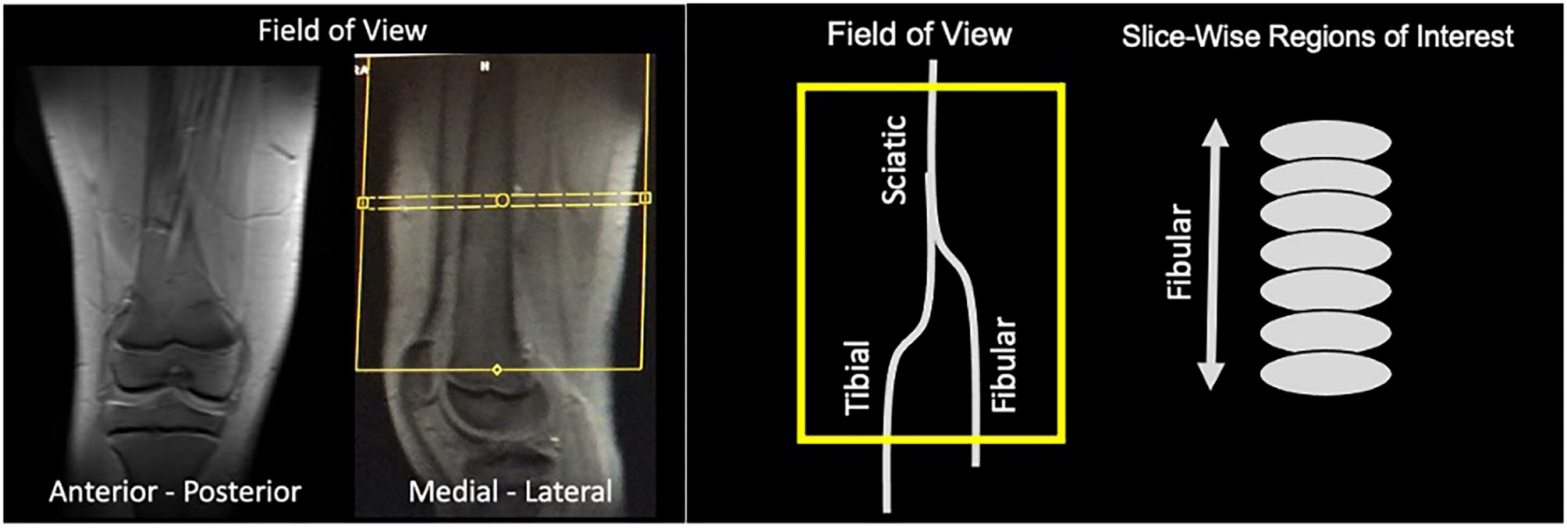
Field of view. Images of an exemplar subjects are shown demonstrating the field of view taking from the anterior-posterior and medial-lateral position. Yellow box indicates the field of view of the diffusion and MTR analyses that contains the three nerve divisions evaluated. For each nerve division, we performed slice-wise regions of interest that were drawn on axial slices.

The scans were performed on a 3T Siemens Trio scanner. Imaging was performed using a 15-Channel knee coil. All subjects underwent conventional MRI scans (T1- and T2-weighted scans), reduced Field-of-View DTI, and MTR scans. The field of view was positioned using a standardized location over the knee (10 cm) using the upper border of the patella as an anatomical reference (Figure 1). The total imaging time was ~30 min for DTI and MTR acquisitions for each leg. DTI scanning parameters included: 20 diffusion directions, *b* = 750 s/mm^2^, voxel size = 0.8 × 0.8 × 5 mm^3^, axial slices = 28, TR/TE = 5,200/103 ms, and 3 averages. MTI parameters included: frequency offset = 1,200 Hz, pulse duration = 9,984 μs, voxel size = 1.3 × 0.9 × 5 mm^3^, axial slices = 28, TR/TE = 1,190/4.37 ms, flip angle = 20°, bandwidth = 380 Hz/Px, and 2 averages. On and Off-Resonance frequency pulses were applied with MTR calculated as the mean ratio of MTR-On to Off sequences. Data analysis was performed using Olea SphereTM V2.3 (Olea Medical®). The sciatic nerve was localized using T2-weighted images. The b0 image was segmented using the Olea auto-adjust segmentation tool. Regions of interest were drawn manually on axial slices at the sciatic, tibial and fibular nerves using the segmented b0 diffusion images and the T2 weighted images as reference. All slices from the field of view were divided into either sciatic, tibial or fibular nerve. Fractional anisotropy (FA), mean diffusivity (MD), radial diffusivity (RD), axial diffusivity (AD), were extracted to evaluate the degree of isotropic motion of water within nerve segments, and therein characterize the integrity of white matter architecture (17).

**Figure 2:**
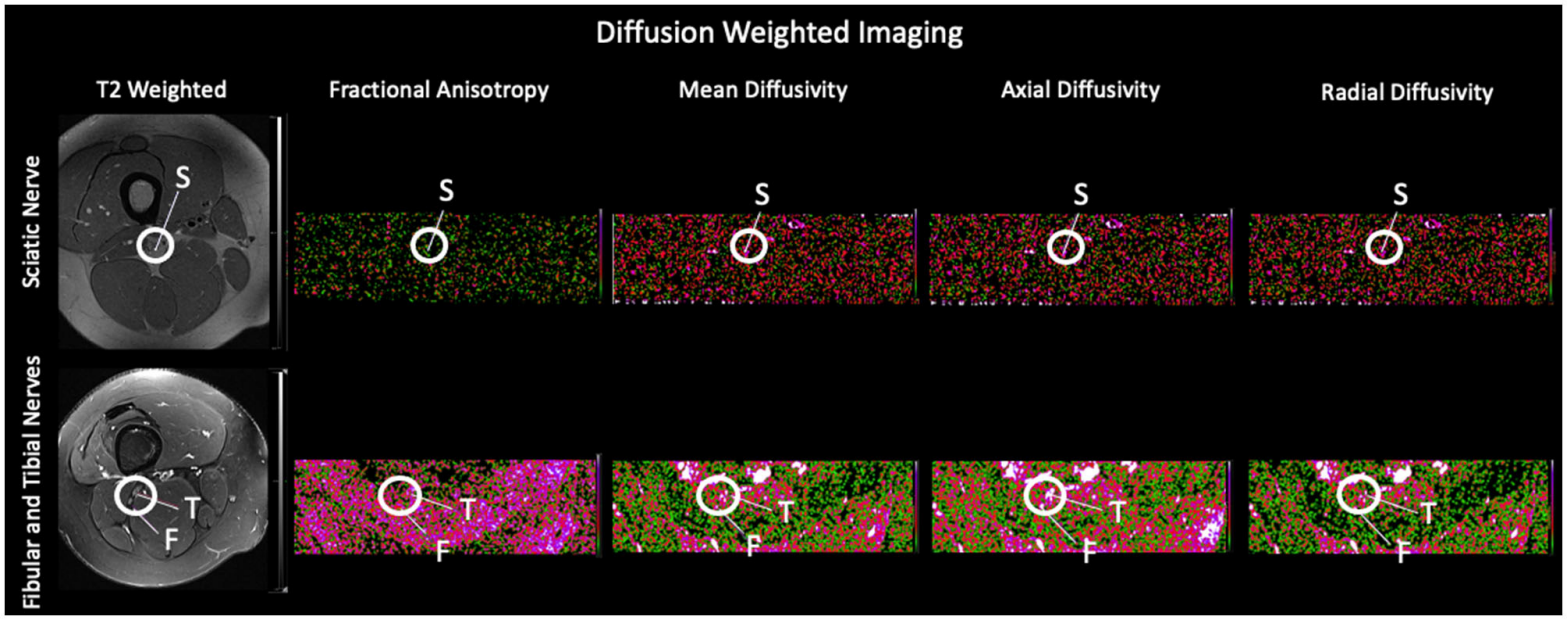
Regions of Interest using Diffusion Tensor Imaging and Magnetization Transfer Ratio Imaging. Exemplar slices from a healthy control are used to show each of the evaluated nerve divisions on the representative T2 weighted scan and their corresponding diffusion weighted scan and using Magnetization Transfer Ratio Imaging. S, Sciatic Nerve; F, Fibular Nerve; T, Tibial Nerve.

Co-registration of b0 diffusion and MTR images was performed and values were reported for each nerve and reflect relative differences in factors such as myelination (14). We collected two independent sequences of DTI and MTI scans. Two-dimensional regions of interest were drawn on each imaging slice using the axial plane within the field of view. Regions of interest are shown for both diffusion weighted imaging and magnetization transfer ratio imaging (Figure 2). Multiple slices were acquired from each nerve bundle above and below the bifurcation of the sciatic nerve into the tibial and fibular divisions. Two metrics were extracted from each region of interest: Mean, reflecting an average of all within ROI values and standard deviation of all values within a given region of interest were calculated.

### Statistical Analysis

Chi-square was used to assess group differences in categorical variables. Multivariate general linear model analysis was conducted using SPSS Statistics. Multivariable regression analysis using Generalized Estimating Equations (GEE) modeling was performed in order to determine the independent association between leg dominance and group (patient vs. control) adjusting for age, sex, number of available scans, and time since injury as covariates. Adjusted differences between patients and controls, 95% confidence intervals and *p*-values are presented. We also performed ROC analyses and provide AUC values when comparing nerve fiber parameters between injured nerve fibers and contralateral controls, and health control values. Correlation analysis was assessed between level of pain reported on the Pain Detection Questionnaire, time since injury and nerve fiber integrity (DTI and MTR metrics). Stata version 16 was used for statistical analysis (Stata Corp LLC, College Station, Texas). Two-tailed *p* < 0.05 with Bonferroni adjustment was used as the criteria for statistical significance (21).

## Results

Group demographics are outlined in Table 1. There was no group difference in terms of age *t*_(53)_ = 1.128, *p* = 0.265, or in the representation of males and females in the two groups, chi square = 0.188, *p* = 0.66. All participants were deemed to have clinically confirmed neuropathic pain from the study physician. Ankle injury was the result of sport, and non-sport related events and did not exceed a grade 2 injury (i.e., there were no complete avulsions). There was a significant increase in reporting of pain symptoms on the Pain Detection Questionnaire for the ankle injury cohort, *t*_(52)_ = 8.93, *p* < 0.001.

**Table 1:** Descriptive statistics.

		**Control**	**Ankle injury**
**Biological sex**			
	Male	8	17
	Female	8	22
**Age**			
		17.56 (4.15)	16.36 (3.35)
**Ankle injury status**			
	None	16	0
	Right (inversion /evervsion)	0	21 (8/4)
	Left (inversion /eversion)	0	18 (10/5)
**Time since injury (days)**			
		-	31 (IQR: 24; Range: 13–92)
Pain detection questionnaire		0.56 (0.96)	6 (IQR: 5; Range: 0–19)

*Mean values are presented alongside standard deviations for group demographics and the Pain Detection Questionnaire. Inv, Inversion; Eve, Eversion*.

**Figure 3:**
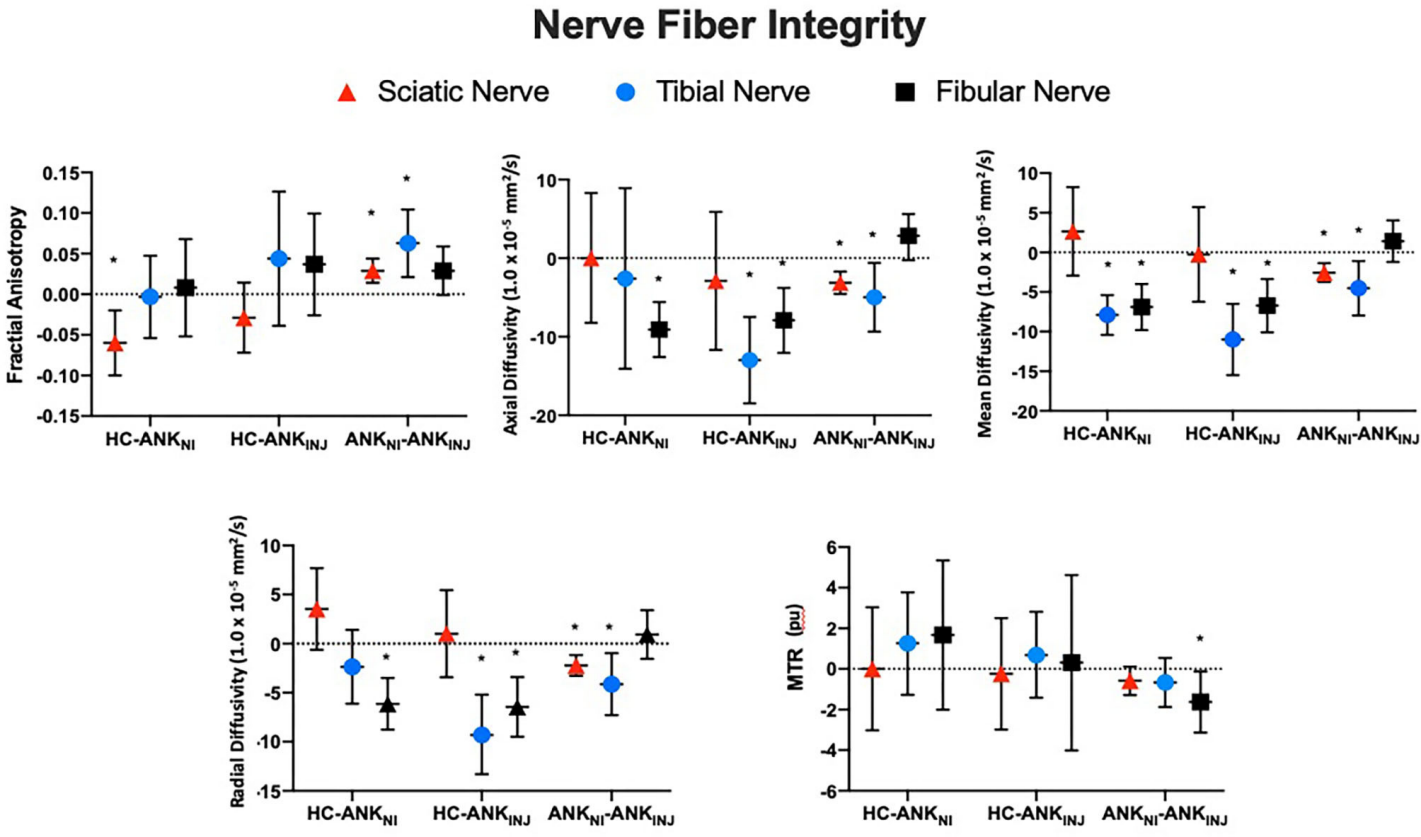
Nerve fiber integrity using adjusted difference scores. Findings from the three nerve fiber divisions (Fibula, Tibia, Sciatic) are presented for each cohort (Healthy, Ankle – Injury, Ankle – No Injury) in terms of the within slice average values. Error bars reflect standard deviations. *Denotes statistical significance for the between group comparison. MTR, Magnetization Transfer Ratio Imaging; HC, Healthy Control; ANK_NI_, Ankle No Injury; ANK_INJ_, Ankle Injury.

The total number of regions of interest were 3,335 which was divided into Sciatic (*n* = 1,636), Tibial (*n* = 848) and Fibial (*n* = 851). Group contrasts for each nerve division are outlined in Table 2 and Figure 3. Comparing healthy controls relative to non-injured legs showed differences in FA, as well as mean and axial diffusivity. Diffusivity findings from the injured leg of ankle injury participants were found to be significantly different from healthy controls in terms of mean, radial, and axial diffusivity (*p* < 0.001). Changes in diffusion and MTR metrics were observed when comparing non-injured with injured legs within the ankle injury cohort. Greater levels were found for FA (Sciatic, Tibial) as well as variance in FA (Sciatic, Tibial, Fibular), Radial (Fibular), Axial (Fibular), and Mean (Fibular) diffusivity. Measures showing higher levels in the injured relative to non-injured legs were found for Radial (Sciatic, Tibial), Axial (Sciatic, Tibial) and Mean (Sciatic, Tibial) diffusivity and MTR (Fibula) as well as variance in Axial diffusivity (Tibial) and MTR (Tibial). Exploring the effect of biological sex showed that greater mean levels were found in tibial and fibular nerve division for radial, axial and mean diffusivity in males than females (*p* < 0.001) as well as greater within slice variance in females (*p* = 0.032).

**Table 2:** Peripheral nerve data.

		**Sciatic nerve**			**Tibial nerve**			**Fibular nerve**		
		**HC – AN_**NI**_**	**HC – AN_**INJ**_**	**AN _**NI**_ – AN _**INJ**_**	**HC – AN_**NI**_**	**HC – AN_**INJ**_**	**AN _**NI**_ – AN _**INJ**_**	**HC – AN_**NI**_**	**HC – AN_**INJ**_**	**AN _**NI**_ – AN _**INJ**_**
**Diffusion tensor imaging**										
FA_M	AD	−0.06 ^**^	−0.029	0.029^***^	−0.003	0.044	0.063^*^	0.008	0.037	0.029
	(CI)	(−0.1, −0.02)	(−0.072, 0.015)	(0.014, 0.044)	(−0.054, 0.047)	(−0.039, 0.126)	(0.021, 0.104)	(−0.052, 0.068)	(−0.026, 0.099)	(−0.001, 0.059)
FA_SD	AD	−0.009	0.005	0.009^**^	−0.009	0.014	0.019^*^	−0.008	0.015	0.023^*^
	(CI)	(−0.024, 0.006)	(−0.008, 0.018)	(0.002, 0.015)	(−0.032, 0.015)	(−0.023, 0.052)	(0.001, 0.037)	(−0.039, 0.022)	(−0.021, 0.05)	(0.009, 0.039)
RD_M	AD	3.53	1.02	−2.22^***^	−2.36	−9.3^***^	−4.13^*^	−6.13^***^	−6.44^***^	0.92
	(CI)	(−0.64, 7.69)	(−3.42, 5.45)	(−3.29, −1.15)	(−6.12, 1.4)	(−13.3, −5.2)	(−7.28, −0.96)	(−8.77, −3.49)	(−9.49, −3.4)	(−1.55, 3.4)
RD_SD	AD	−50.8	−52.5	−0.7	−1.53	−1.17	−1.04	−12.7	−9.97	1.87 ^*^
	(CI)	(−126.6, 25)	(−130.6, 25.6)	(−1.44, 0.03)	(−4.58, 1.53)	(−5.67, 3.33)	(−2.6, 0.52)	(−29.9, 4.57)	(−31.5, 11.6)	(0.47, 3.27)
AD_M	AD	0.014	−2.9	−3.14^***^	−2.6	−13^***^	−4.99^*^	−9.1^***^	−7.91^***^	2.84
	(CI)	(−8.25, 8.28)	(−11.7, 5.9)	(−4.57, −1.72)	(−14.1, 8.92)	(−18.5, −7.5)	(−9.37, −0.62)	(−12.6, −5.6)	(−12.05, −3.78)	(−0.27, 5.6)
AD_SD	AD	−91.4	−84.2	0.05	−3.13	−3.26	−2.32^*^	−146	−179	2.82^***^
	(CI)	(−226, 43.8)	(−220.5, 52)	(−0.77, 0.87)	(−7.06, 0.81)	(−8.64, 2.12)	(−4.44, −0.2)	(−448, 156)	(−553, 194)	(1.11, 4.53)
MD_M	AD	2.65	−0.26	−2.55^***^	−7.89^***^	−11.0^***^	−4.52^*^	−6.9^***^	−6.72^***^	1.41
	(CI)	(−2.94, 8.24)	(−6.23, 5.7)	(−3.73, −1.37)	(−10.4, −5.39)	(−15.5, −6.5)	(−7.96, −1.08)	(−9.8, −4)	(−10.08, −3.37)	(−1.21, 4.03)
MD_SD	AD	−0.43	−0.43	−0.47	−1.9	−1.65	−1.46	−101	−125	2.2^**^
	(CI)	(−3.05, 2.2)	(−2.8, 1.93)	(−1.21, 0.27)	(−5.11, 1.31)	(−6.22, 2.91)	(−3.1, 0.19)	(−315, 112)	(−390, 140)	(0.73, 3.68)
**Magnetization transfer ratio**										
MTR_M	AD	0.008	−0.24	−0.58	1.26	0.69	−0.67	1.67	0.31	−1.63^*^
	(CI)	(−3.03, 3.04)	(−2.98, 2.5)	(−1.28, 0.11)	(−1.27, 3.78)	(−1.42, 2.81)	(−1.88, 0.54)	(−2.01, 5.34)	(−4.02, 4.63)	(−3.14, −0.13)
MTR_SD	AD	0.019	−0.03	0.21	−0.34	−0.34	−1.0^*^	−1.52	−0.89	−0.09
	(CI)	(−2.24, 2.28)	(−2.38, 2.31)	(−0.24, 0.66)	(−2.75, 2.08)	(−2.9, 2.23)	(−1.84, −0.16)	(−3.54, 0.5)	(−2.06, 1.27)	(−0.95, 0.78)

*M, mean values within imaging slices and SD reflects standard deviation within a slice. Groups are compared based on diffusion tensor imaging and magnetization transfer ratio imaging with adjusted difference values reported*.

* *p < 0.05*,

** *p < 0.005*,

*** *p < 0.001*.

*HC, Healthy Control; AN_NI_, Ankle No Injury; AN_INJ_, Ankle Injury*.

**Figure 4:**
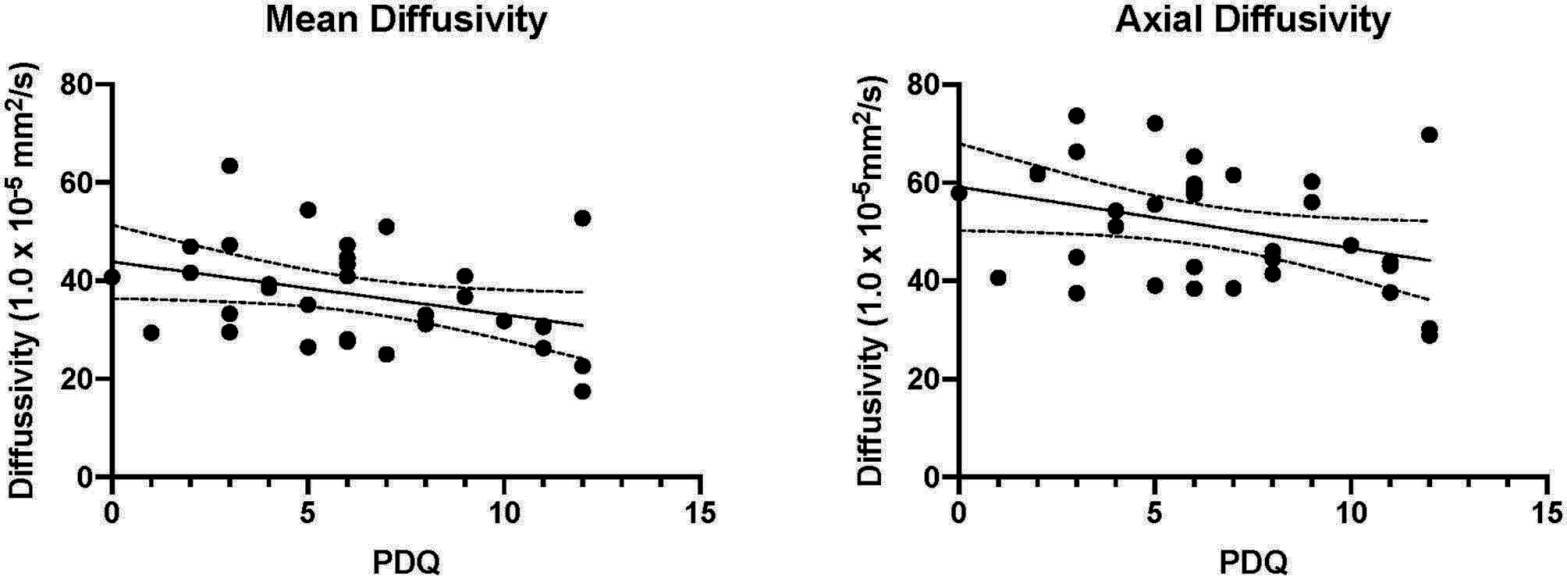
Correlations between pain reporting and imaging. The significant correlations are plotted between axial and mean diffusivity of the injured fibular nerve. PDQ, Pain Detection Questionnaire.

Correlation analysis showed that the PDQ scores were correlated with the AD (*r* = 0.176, *p* = 0.389) and MD (*r* = 0.234, *p* = 0.250) from the fibial nerve (Figure 4). However, both correlations did not survive after controlling for time since injury (AD: *r* = 0.176, *p* = 0.389; MD: *r* = 0.234, *p* = 0.250). Findings from the AUC analysis showed that the highest AUCs were found when comparing a participants injured leg to their contralateral control with the highest value found for FA in the Fibial Nerve (see Table 3).

**Table 3:** Area under the curve.

	**Injured nerve to contralateral control**			**Injured nerve to healthy control**		
	**Fibial**	**Tibial**	**Sciatic**	**Fibial**	**Tibial**	**Sciatic**
Fractional anisotropy	0.665	0.576	0.563	0.532	0.546	0.41
Radial diffusivity	0.402	0.422	0.377	0.456	0.406	0.565
Axial diffusivity	0.45	0.438	0.377	0.462	0.452	0.542
Mean diffusivity	0.418	0.423	0.373	0.448	0.404	0.567
Magnetization transfer ratio	0.492	0.503	0.516	0.513	0.542	0.519

*The AUC was calculated for comparing a participatns injured leg relative to their contralateral control and for comparing an injured nerve to a healthy control participant. Values are provided for the mean data for DTI and MTR metrics*.

**Figure 5:**
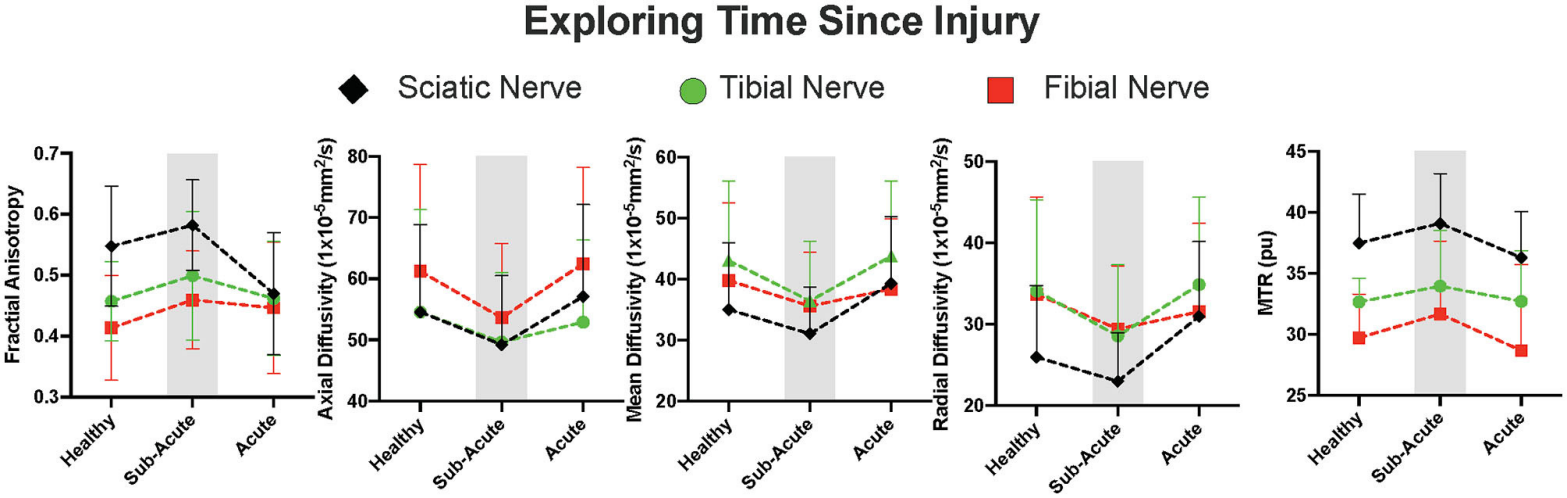
Time since injury and nerve fiber integrity. An exploratory and qualitative analysis was performed where ankle injury participants were divided into those who were within 30 days from injury (sub-acute; gray panels) and those between 30 days and 3 months (acute) highlighting a sub-acute phase of nerve fiber damage. Reported data reflect average within slice values for each imaging modality.

We performed an exploratory analysis using time since injury to understand temporal variation in our acute time window. As is shown in Figure 5, all imaging variables appeared to show a time-sensitive pattern. This was found for both diffusion weighted and magnetization transfer imaging variables where in a sub-acute (<30 days) time window, axial, radial and mean diffusivity all decreased and then return to levels approximating that of healthy controls. Opposite findings were found for FA as well as MTR values.

## Discussion

In prior investigations, we demonstrated imaging changes of the diffuse nature of neuropathic pain in the PNS and CNS (4). Here we extend these findings in a mixed cohort of patients with left and right ankle injury. The main findings were (1) changes in nerve fiber integrity evinced by DTI and MTR in ipsi- and contralateral nerve fibers to the site of injury, (2) a correlation between DTI from the injured fibular nerve and PDQ, and (3) a pattern of changes in nerve fiber integrity suggesting a sub-acute time window for nerve pathology.

### Peripheral Fiber Neurography

Ankle injury participants were confirmed to be in a neuropathic pain state based on a clinical examination that used one or more features of neuropathic pain (22). Comparing data from the affected limb to healthy controls demonstrated group diffrences in the tibial and fibular nerve divisions. All of these comparisons reflected greater levels of RD, MD, and AD in the affected limb suggesting a less restricted diffusion environment. This aligns with observations of inflammation after injury (23, 24) and the degenerative phase following peripheral nerve injury (25). Comparing the affected vs. unaffected limb, a similar trend is observed where the affected limb has less restricted diffusion. Notably, the fibular nerve showed greater MTR levels in the affected vs. unaffected limb, suggesting greater tissue presence in the former that may be the result of fibrotic thickening with scar tissue formation (26) rather than remodeling which would be seen with a decrease in MTR (27). It is notable that findings from this nerve fiber division also showed a unique trend with greater variability in within slice AD, RD, and MD, again possibly owing to local heterogeneity in cellular composition. AUC analyses also underscore the fibular nerve as the region with greatest potential to differentiate cohorts, with highest levels coming from comparisons with contralateral nerves, rather than nerve fiberes from healthy controls. Importantly, these values are relatively low overall which may be impacted by the current sample size. Interestingly, there were some group differences between healthy controls and the unaffected leg. As shown in patients with complex regional pain syndrome, nerve pathology can be diffuse and so bilateral nerve fiber changes are possible [e.g., (28)]. However, it may be that these simply reflect inter-subject differences that do not reflect a pathological state, or the result of a biomechnical adaptation where patients rely more heavily on the unaffected leg. Based on earlier observations from our lab (4) intra-subject comparisons may reflect non-pathological processes such as dominance of nerve fibers, which was controlled for alongside other known mitigating variables. As such, we suggest that findings of group differences in the non-affected side either reflect a progression of neuropathic pain or form of biomechnical adaptation that should be confirmed in future longitudinal work.

### Objective (Nerve Changes) and Subjects (Pain) Measures

Clinical features of neuropathic pain represent a broad disregulation of normal sensory functioning (22). Features such as allodynia and loss of sensation can be present and are captured in the PDQ which is a standard clinical tool for assessing neuropathic pain. In the evaluated cohort, patients showed elevated reporting on the PDQ, consistent with an increased reporting of pain stemming from their ankle (left or right) injury. To minimize the number of statistical comparisons, participant scores on the PDQ were correlated with nerve fiber integrity from the affected (rather than unaffected) leg as the injury site is where PDQ scores were scored. Significant correlations were observed in the fibular nerve of the injured legs for AD and MD and self-reported pain levels (i.e., PDQ). Although both correlations were relatively weak and no longer significant after controlling for time since injury, it is interesting to note that the nerve specificity may correspond with the more frequent reporting of inversion sprains that would put tension on the nerves innervating the lateral component of the ankle (i.e., fibular division). Moreover, a negative correlation between MD and AD with PDQ scores may highlight the presence of axonal damage that restricts water diffusion (29). As to why we didn't observe more correlations between nerve fiber characteristics and pain reporting we present three explanations. First, the use of psychometrics relies heavily on accurate self-reporting as well as test sensitivity and specficity. Prior reports on the sensitivity and specificity for the PDQ have shown a relatively high level of performance with 85 and 80%, respectively (28), which should be factored into any interpretation. Second, findings may reflect the presence of sub-clinical levels of pathology. That is, there may be sufficient pathology to observe differences in nerve fiber integrity; however, insufficient to drive active pain reporting. We have shown in a previous publication (4) that peripheral nerve fiber pathology is associated with changes in brain structure and function suggesting that some degree of central sensitization (1) may have occurred in the current cohort. Thirdly, the variable time since injury may reflect different periods that have distinct relationships between the extent of pathology and behavior. We explore this further in the next section. Together, findings support the presence of altered nerve fiber integrity in persons with clinically confirmed neuropathic pain that may correlate with self-reported pain levels. These findings provide support for the application of peripheral nerve imaging (1) in a neuropathic pain diagnosis, (2) in clinical populations with difficulty expressing pain symptoms, and (3) to understand the neurobiological basis of pain progression and adaptation throughout the nervous system.

### Temporal Measures: Nerve Metrics

Participants were on average 37 days after their injury which ranged from 13 to 92 days. After observing that pain reporting was significantly and negatively correlated with time since injury we divided our ankle injury cohort into an acute (>30 days since injury) and sub-acute (<30 days since injury) group (see Figure 5). This timeline is notable as a peak in nerve permeability has been observed around the 4–7 day period as the peak of the acute inflammatory response (30, 31) with a second spike in permeability around 4 weeks after transection in mice (30). As such, this assigned time window should not obscure biological windows of the inflammatory response resulting from wallerian degeneration. From this analysis, we can observe three unique features. First, the sub-acute window appears to present a transitory, period with unique nerve fiber characteristics from diffusion and MTI data. Second, there is evidence of increased restriction from axial, radial and mean diffusivity which may align with the increase influx in calcium ions during wallerian degeneration targetted at sealing axons that have been damaged (32). Thirdly, we see evidence of changes in the relative concentration of water and a semi-solid molecules (e.g., myelin) (33), which likely reflects a decrease in available water in the area from the changes in nerve permeability. This would align as well with the findings from FA, where greater relative restriction in the secondary and tertiary eigen vectors (contributing toward radial diffusivity) than the primary (axial diffusivity) will result in an increase in FA. Notably, the timecourse of resolution is mediated by a highly complex series of events (34, 35), and the current analysis was not powered to objectively evaluate this research question. Future work more focused on longitudinal monitoring of patients is required to evaluate this hypothesis.

### Caveats

There are a few caveats to consider: (1) *Inter- and intra subject variability in nerve fiber characteristics:* One major caveat of comparision of affected vs. unaffected legs is that there is a natural laterality of nerve differences between dominant and non dominant limbs. This was controlled for statistically in the current investigation; however, it is possible that other natural source of variability occur. It is important to establish normative data sets for such reasons. (2) Participant heterogeneity: The timeline of injury with the included patient cohort varied from 15 to 110 days and was focused on the “Acute” time window. Notably, this may still have included significant variability in the underlying group comparisons. Evidence for this was found in *post-hoc* analysis showing how diffusion and MTR metrics varied as a function of time. It will be imperative for future research to draw shorter time windows and develop participant specific models for nerve fiber resolution. (3) Body mass index. The current study did not include any measure of BMI. This would have allowed furthe interpetation of peripheral nerve changes and the adaptive or maladaptive characteristics. Future research will include BMI for all participants. *Temporal changes*: In our cohort we could define the timeline for the injury; however, we lacked within participant temporal data that could inform discussion on resolution or persistence of changes in nerve fiber integrity. This work is currently underway in our laboratory. (4) *Effect of Medications:* Most patients were on some form of medication acutely; however, our sample size did not permit accurate evaluation of the impact of medication on nerve fiber characteristics. (5) MTR sequences. Current sequences did not perform fat saturation which may have greater impact on smaller nerve fibers like the tibial and fibular nerves than larger bundles including the sciatic. Use of newer sequences that integrate fat saturation may help improve image quality (6) Sample size. The current study was part of a larger study evaluating central and peripheral nervous system changes in persons with neuropathic pain. The healthy control cohort was smaller than the clinical cohort because we anticipated a relatively lower level of variance in DTI and MTR attributable to the absence of trauma. In the current investigation, we were able to achieve a power of 0.67; however, larger cohorts in future investigations may increase this power to evaluate temporal characteristics of nerve fiber changes and improve AUC analysis.

### Conclusions

Peripheral nerve fiber integrity is compromised and shows evidence of a more restricted diffusion environment in persons with clinically confirmed neuropathic pain. Findings showed evidence to suggest the presence of different patterns of nerve repair including scar tissue formation and a sub-acute time window where all DTI and MTR variables showed similar patterns of variability. The application of magnetic resonance neurography to evaluate patients with suspected neuropathy is reliable and highlights differences that may inform clinical evaluation. Future investigations aimed at improving clinical decision making will benefit by integrating peripheral neurography with central measures of nociception and pain perception.

## Data Availability Statement

The original contributions presented in the study are included in the article/supplementary material, further inquiries can be directed to the corresponding author.

## Ethics Statement

The studies involving human participants were reviewed and approved by Boston Children's Hospital Institutional Review Board. Written informed consent to participate in this study was provided by the participants' legal guardian/next of kin.

## Author Contributions

SH, DZ, RB, AL, LS, and DB contributed to conception and design of the study. AK organized the database and performed participant recruitment. SS and DZ performed the statistical analysis. SH wrote the first draft of the manuscript. All authors contributed to manuscript revision, read, and approved the submitted version.

## Funding

This work was supported by the National Institutes of Health (R01HD083133-01A1). This funding was provided to cover participant recruitment, equipment use, and salary as well as open access fees for publication of research findings.

## Conflict of Interest

The authors declare that the research was conducted in the absence of any commercial or financial relationships that could be construed as a potential conflict of interest.

## Publisher's Note

All claims expressed in this article are solely those of the authors and do not necessarily represent those of their affiliated organizations, or those of the publisher, the editors and the reviewers. Any product that may be evaluated in this article, or claim that may be made by its manufacturer, is not guaranteed or endorsed by the publisher.

## References

[1] Ji R-R Nackley A Huh Y Terrando N. Maixner W. Neuroinflammation and central sensitization in chronic and widespread pain. Anesthesiology. (2018) 129:343–66. 10.1097/ALN.0000000000002130 29462012 PMC6051899

[2] Haythornthwaite J Benrud-Larson L. Psychological aspects of neuropathic pain. Clin J Pain. (2000) 16:S101–5. 10.1097/00002508-200006001-00017 10870748

[3] Colloca L Ludman T Bouhassira D Baron R Dickenson AH Yarnitsky D. Neuropathic pain. Nat Rev Dis Primer. (2017) 3:17002. 10.1038/nrdp.2017.2 28205574 PMC5371025

[4] Holmes S Barakat N Bhasin M Lopez NI Lebel A Zurakowski D. Biological and behavioral markers of pain following nerve injury in humans. Neurobiol Pain. (2020) 7:100038. 10.1016/j.ynpai.2019.100038 31890990 PMC6926375

[5] Jensen TS Finnerup NB. Allodynia and hyperalgesia in neuropathic pain: clinical manifestations and mechanisms. Lancet Neurol. (2014) 13:924–35. 10.1016/S1474-4422(14)70102-4 25142459

[6] Chiaramonte R Romano M Vecchio M. A Systematic review of the diagnostic methods of small fiber neuropathies in rehabilitation. Diagnostics. (2020) 10:613. 10.3390/diagnostics10090613 32825514 PMC7554909

[7] Ackerley R Watkins RH. Microneurography as a tool to study the function of individual C-fiber afferents in humans: responses from nociceptors, thermoreceptors, and mechanoreceptors. J Neurophysiol. (2018) 120:2834–46. 10.1152/jn.00109.2018 30256737

[8] Mellgren SI Nolano M Sommer C. The cutaneous nerve biopsy. In: Handbook of Clinical Neurology. Elsevier (2013). p. 171–88. Available online at: https://linkinghub.elsevier.com/retrieve/pii/B9780444529022000102 (accessed May 24, 2021). 10.1016/B978-0-444-52902-2.00010-2 23931780

[9] Menorca RMG Fussell TS Elfar JC. Nerve physiology. Hand Clin. (2013) 29:317–30. 10.1016/j.hcl.2013.04.002 23895713 PMC4408553

[10] Chen Y Haacke EM Li J. Peripheral nerve magnetic resonance imaging. F1000Research. (2019) 8:1803. 10.12688/f1000research.19695.1 31700612 PMC6820826

[11] Hiltunen J Suortti T Arvela S Seppä M Joensuu R Hari R. Diffusion tensor imaging and tractography of distal peripheral nerves at 3 T. Clin Neurophysiol. (2005) 116:2315–23. 10.1016/j.clinph.2005.05.014 16125460

[12] Simon NG Lagopoulos J Gallagher T Kliot M Kiernan MC. Peripheral nerve diffusion tensor imaging is reliable and reproducible: reliability of peripheral nerve DTI. J Magn Reson Imaging. (2016) 43:962–9. 10.1002/jmri.25056 26397723

[13] Boyer RB Kelm ND Riley DC Sexton KW Pollins AC Shack RB. et al. 47-T diffusion tensor imaging of acute traumatic peripheral nerve injury. Neurosurg Focus. (2015) 39:E9. 10.3171/2015.6.FOCUS1590 26323827 PMC4786003

[14] Schmierer K Scaravilli F Altmann DR Barker GJ Miller DH. Magnetization transfer ratio and myelin in postmortem multiple sclerosis brain. Ann Neurol. (2004) 56:407–15. 10.1002/ana.20202 15349868

[15] Levy D Gruener H Riabinin M Feingold Y Schreiber S Pick CG. Different clinical phenotypes of persistent post-traumatic headache exhibit distinct sensory profiles. Cephalalgia. (2019) 17:033310241989636. 10.1177/0333102419896368 31847569 PMC10589814

[16] Kollmer J Kästel T Jende JME Bendszus M Heiland S. Magnetization transfer ratio in peripheral nerve tissue: does it depend on age or location? Invest Radiol. (2018) 53:397–402. 10.1097/RLI.0000000000000455 29470194

[17] Anderson RB Hunt KJ. McCormick JJ. Management of Common Sports-related Injuries About the Foot and Ankle. Am Acad Orthop Surg. (2010) 18:546–56. 10.5435/00124635-201009000-00006 20810936

[18] Pommering T Kluchurosky L Hall S. Ankle and foot injuries in pediatric and adult athletes. Prim Care Clin Off Pract. (2005) 32:133–61. 10.1016/j.pop.2004.11.003 15831316

[19] Baima J Krivickas L. Evaluation and treatment of peroneal neuropathy. Curr Rev Musculoskelet Med. (2008) 1:147–53. 10.1007/s12178-008-9023-6 19468889 PMC2684217

[20] Freynhagen R Baron R Gockel U Tölle TR. Pain DETECT : a new screening questionnaire to identify neuropathic components in patients with back pain. Curr Med Res Opin. (2006) 22:1911–20. 10.1185/030079906X132488 17022849

[21] Staffa SJ Zurakowski D. Strategies in adjusting for multiple comparisons: a primer for pediatric surgeons. J Pediatr Surg. (2020) 55:1699–705. 10.1016/j.jpedsurg.2020.01.003 32029234

[22] Baron R Binder A Wasner G. Neuropathic pain: diagnosis, pathophysiological mechanisms, and treatment. Lancet Neurol. (2010) 9:807–19. 10.1016/S1474-4422(10)70143-5 20650402

[23] Alexander JJ Anderson AJ Barnum SR Stevens B Tenner AJ. The complement cascade: Yin-Yang in neuroinflammation - neuro-protection and -degeneration. J Neurochem. (2008) 107:1169–87. 10.1111/j.1471-4159.2008.05668.x 18786171 PMC4038542

[24] Chen Y-Y Zhang X Lin X-F Zhang F Duan X-H Zheng C-S. DTI metrics can be used as biomarkers to determine the therapeutic effect of stem cells in acute peripheral nerve injury: DTI of stem cell therapy in nerve injury. J Magn Reson Imaging. (2017) 45:855–62. 10.1002/jmri.25395 27448779

[25] Yamasaki T Fujiwara H Oda R Mikami Y Ikeda T Nagae M. *In vivo* evaluation of rabbit sciatic nerve regeneration with diffusion tensor imaging (DTI): correlations with histology and behavior. Magn Reson Imaging. (2015) 33:95–101. 10.1016/j.mri.2014.09.005 25271136

[26] Kajita Y Suetomi K Okada T Ikeuchi M Arai Y-C Sato K. Behavioral and neuropathological changes in animal models of chronic painful scar. J Orthop Sci. (2013) 18:1005–11. 10.1007/s00776-013-0453-7 23963587 PMC3838578

[27] Liu Z Pardini M Yaldizli Ö Sethi V Muhlert N Wheeler-Kingshott CAM. Magnetization transfer ratio measures in normal-appearing white matter show periventricular gradient abnormalities in multiple sclerosis. Brain. (2015) 138:1239–46. 10.1093/brain/awv065 25823475 PMC5963416

[28] Palmer S Bailey J Brown C Jones A McCabe CS. Sensory function and pain experience in arthritis, complex regional pain syndrome, fibromyalgia syndrome, and pain-free volunteers: a cross-sectional study. Clin J Pain. (2019) 35:894–900. 10.1097/AJP.0000000000000751 31408010

[29] Winklewski PJ. Understanding the physiopathology behind axial and radial diffusivity changes-what do we know? Front Neurol. (2018) 9:6. 10.3389/fneur.2018.00092 29535676 PMC5835085

[30] Mizisin AP Weerasuriya A. Homeostatic regulation of the endoneurial microenvironment during development, aging and in response to trauma, disease and toxic insult. Acta Neuropathol (Berl). (2011) 121:291–312. 10.1007/s00401-010-0783-x 21136068 PMC3038236

[31] Weerasuriya A Hockman CH. Perineurial permeability to sodium during Wallerian degeneration in rat sciatic nerve. Brain Res. (1992) 581:327–33. 10.1016/0006-8993(92)90727-Q 1466671

[32] Tsao JW George EB Griffin JW. Temperature modulation reveals three distinct stages of wallerian degeneration. J Neurosci. (1999) 19:4718–26. 10.1523/JNEUROSCI.19-12-04718.1999 10366605 PMC6782642

[33] Vavasour IM Laule C Li DKB Traboulsee AL MacKay AL. Is the magnetization transfer ratio a marker for myelin in multiple sclerosis? J Magn Reson Imaging. (2011) 33:710–8. 10.1002/jmri.22441 21563257

[34] Omura K Ohbayashi M Sano M Omura T Hasegawa T Nagano A. The recovery of blood-nerve barrier in crush nerve injury-a quantitative analysis utilizing immunohistochemistry. Brain Res. (2004) 1001:13–21. 10.1016/j.brainres.2003.10.067 14972650

[35] Powell HC Myers RR Costello ML Lampert PW. Endoneurial fluid pressure in wallerian degeneration. Ann Neurol. (1979) 5:550–7. 10.1002/ana.410050610 475350

